# The complete mitochondrial genome of *Aglais ladakensis* (Lepidoptera: Nymphalidae: Nymphalinae)

**DOI:** 10.1080/23802359.2019.1711224

**Published:** 2020-01-16

**Authors:** Keke Chen, Chengcai Si, Zhongqi Pan, Jiasheng Hao

**Affiliations:** Laboratory of Molecular Evolution and Biodiversity, College of Life Sciences, Anhui Normal University, Wuhu, P. R. China

**Keywords:** Mitochondrial genome, phylogeny, Nymphalinae, Nymphalini, *Aglais ladakensis*

## Abstract

We describe the mitogenome sequence of alpine butterfly *Aglais ladakensis* (Lepidoptera: Nymphalidae: Nymphalinae) collected from the Qilianshan Mountain, Gansu province, China. The molecule is 15,222 bp in length, containing 37 typical insect mitochondrial genes and one AT-rich region. All protein-coding genes (PCGs) start with ATN codons, except for *COI* gene with CGA, which is often found in other butterflies. In addition, seven PCGs harbor the typical stop codon TAA, whereas six PCGs terminate with TA or T. The *rrnL* and *rrnS* genes are 1316 bp and 735 bp in length, respectively. The AT-rich region is 394 bp in size and harbors several features characteristic of the lepidopterans, including the motif ATAGA followed by a 19 bp poly-T stretch and a microsatellite-like (TA)_8_ element. Phylogenetic analysis shows that the Qinghai-Tibet Plateau (QTP) distributed *A. ladakensis* of this study is closely related to the *A. milberti*, which is the only *Aglais* species that occurs in the alpine caves of North America.

The tribe Nymphalini is one of the most diverse butterfly groups distributed all around the world and this diversity (e.g. wing patterns, behaviors, and host-plant associations) have led to many Nymphalini species as model organisms in evolutionary and ecological studies (Janz et al. 2001; Wahlberg et al. 2005). However, the taxonomy and phylogeny of Nymphalini are still standing as a controversial issue (Harvey 1991; Wahlberg 2006). In recent decades, mitogenome have been widely used as an informative molecular marker in evolutionary study areas (Boore 1999; Galtier et al. 2009).

In this study, we newly determined the complete mitogenome sequence of the *Aglais ladakensis*, and reconstructed the phylogenetic tree of the species with other related Nymphalini species. *Aglais ladakensis* (distributed at about 4200 meters high) was collected from Qilianshan Mountain, Qinhai province, China (coordinates: E100.39, N38.50) in July 2016. After morphological identification, a voucher specimen (ANUH-20160710) was kept in the Herbarium of Anhui Normal University (Wuhu, China). Total genomic DNA was extracted from thorax muscle using a DNA extraction kit (Shanghai, China) and the PCR amplication and sequencing were conducted after Hao et al. (2013). The resultant reads were assembled and annotated using the BioEdit 7.0 (Hall 1999) and MEGA 7.0 (Kumar et al. 2016).

The whole mitogenome is a circular molecule of 15,222 bp in size (GenBank accession No. MN732892) with a AT bias of 80.5%, containing 13 protein-coding genes (PCGs), 22 transfer tRNA genes, 2 ribosomal rRNA genes, and a noncoding AT-rich control region. All PCGs are initiated by typical ATN codons, except for *COI* gene, which starts with the unusual CGA as observed in most of the other sequenced nymphalids (Gan et al. 2016; Timmermans et al. 2016). Seven PCGs have a complete stop codon TAA, whereas six PCGs (*COI*, *COII*, *COIII*, *ND1*, *ND4*, and *ND5*) stop with incomplete TA or T. All tRNAs harbor the typical predicted secondary cloverleaf structures except for the tRNA^Ser^(AGN), as seen in other determined butterflies (Hao et al. 2013; Shi et al. 2018). The *rrnL* and *rrnS* genes are 1316 bp and 735 bp in length, respectively. The AT-rich region is 394 bp long with several structures characteristic of lepidopterans, including the motif ATAGA followed by a 19 bp poly-T stretch and a microsatellite-like (TA)_8_ element.

Using two *Baeotus* nymphalid species as the outgroups, the maximum-likelihood (ML) phylogenetic analysis were performed with IQ-TREE v.1.6.8 (Nguyen et al. 2015) based on the concatenated mitochondrial *COI* and two nuclear gene (*EF-1a*, and *wingless*) sequence data to clarify the phylogenetic relationship of *A. ladakensis* with other 50 nymphalids available from GenBank (Figure 1). The resultant phylogenetic tree showed that the Nymphalini of this study includes 12 genera, the genus *Aglais* is sister to the grouping of two genera, namely, the *Nymphalis* and *Polygonia*; in addition, the alpine *A. ladakensis* of this study that is distributed Qinghai-Tibet Plateau (QTP) is closely related to the Milbert's Tortoiseshell *A. milberti*, which is the only *Aglais* species that occurs in the alpine caves of North America.

**Figure 1.**
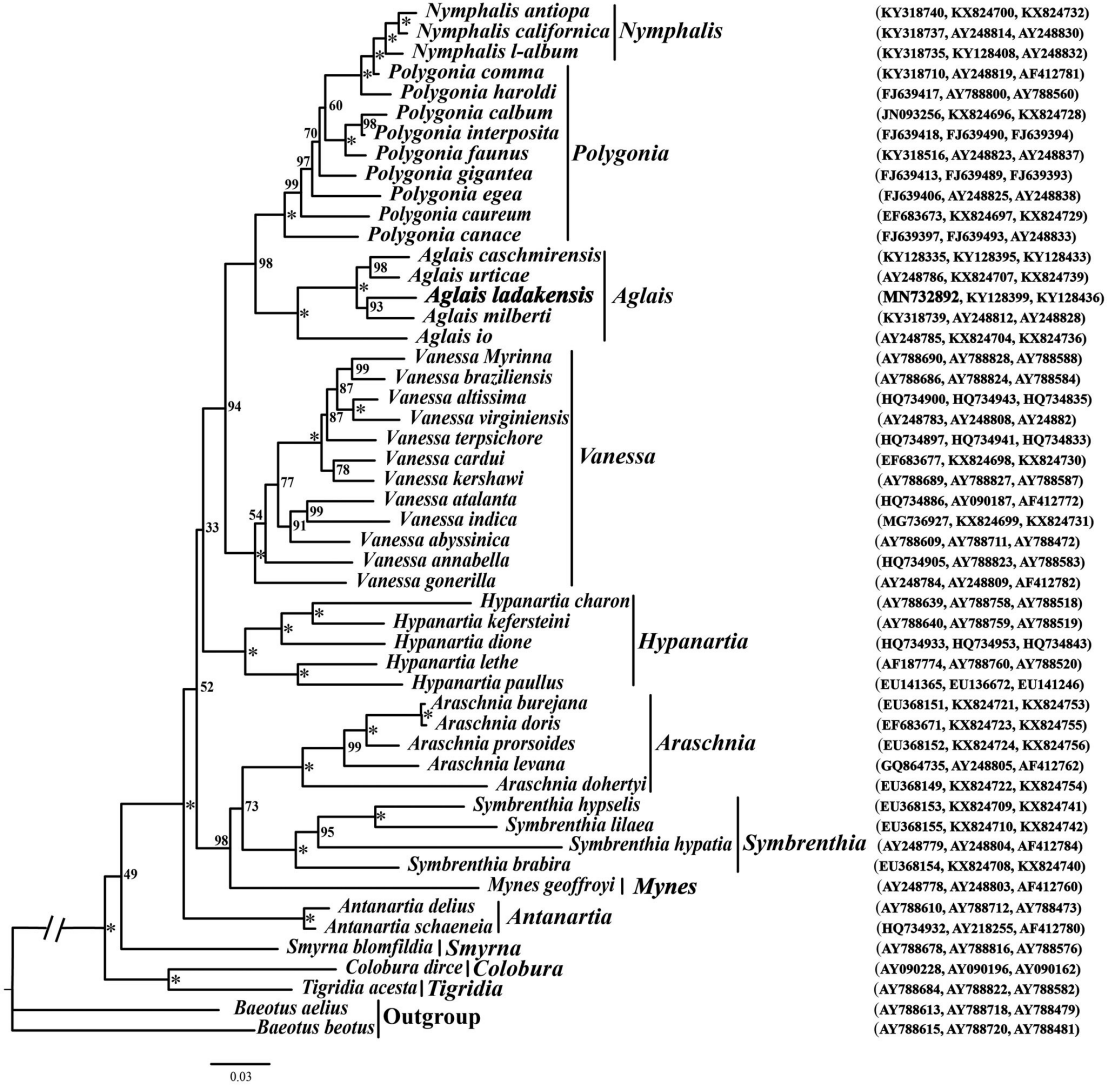
The maximum-likelihood (ML) phylogenetic tree of Nymphalini inferred from nucleotide sequence data of mitochondrial *CO1*, nuclear *EF-1a* and *wingless* genes. The numbers beside the nodes are percentages of 1000 bootstrap values (*BP = 100%). The alphanumeric characters in parentheses represent the GenBank accession numbers.
